# A nomogram for predicting CRT response based on multi-parameter features

**DOI:** 10.1186/s12872-024-04033-4

**Published:** 2024-07-19

**Authors:** Yuxuan Lou, Yang Hua, Jiaming Yang, Jing Shi, Lei Jiang, Yang Yang

**Affiliations:** 1 Southeast University, Nanjing, 210009 Jiangsu China; 2 Department of Cardiology, The First Affiliated Hospital with Nanjing Medical University, Nanjing, 210029 Jiangsu China

**Keywords:** Heart failure, Cardiac resynchronization therapy (CRT), Multiparameter features, Nomogram

## Abstract

**Objective:**

To construct a nomogram for predicting the responsiveness of cardiac resynchronization therapy (CRT) in patients with chronic heart failure and verify its predictive efficacy.

**Method:**

A retrospective study was conducted including 109 patients with chronic heart failure who successfully received CRT from January 2018 to December 2022. According to patients after six months of the CRT preoperative improving acuity in the left ventricular ejection fraction is 5% or at least improve grade 1 NYHA heart function classification, divided into responsive group and non-responsive group. Clinical data of patients were collected, and LASSO regression analysis and multivariate logistic regression analysis were used to explore relative factors. A nomogram was constructed, and the predictive performance of the nomogram was evaluated using the calibration curve and decision curve analysis (DCA).

**Results:**

Among the 109 patients, 61 were assigned to the CRT-responsive group, while 48 were assigned to the non-responsive group. LASSO regression analysis showed that left ventricular end-systolic volume, diffuse fibrosis, and left bundle branch block (LBBB) were independent factors for CRT responsiveness in patients with heart failure (*P* < 0.05). Based on the above three predictive factors, a nomogram was constructed. The ROC curve analysis showed that the area under the curve (AUC) was 0.865 (95% CI 0.794–0.935). The calibration curve analysis showed that the predicted probability of the nomogram is consistent with the actual occurrence rate. DCA showed that the line graph model has an excellent clinical net benefit rate.

**Conclusion:**

The nomogram constructed based on clinical features, laboratory, and imaging examinations in this study has high discrimination and calibration in predicting CRT responsiveness in patients with chronic heart failure.

## Introduction

Chronic heart failure, referred to the gradual appearance of symptoms and signs of heart failure on the basis of original chronic heart diseases, stands as a major cardiovascular cause of hospitalization and mortality. Epidemiological surveys reveal that among individuals aged 35 and above in China, the occurrence of heart failure soars to 1.3% [1]. Cardiac resynchronization therapy (CRT) has emerged as a widely utilized clinical treatment for heart failure, both domestically and internationally [2]. In recent years, CRT has progressed into a practical non-pharmacological approach for chronic heart failure, which could mitigate the desynchronization of the heart’s mechanical contractions, enhance cardiac functionality, and reduce mortality. Nevertheless, the CRT response varies significantly. Despite stringent patient selection based on clinical guidelines, approximately 30% of patients remain non-responsive to CRT [3, 4]. Given the intricacies of CRT procedures and the significant financial burden of treatment, developing an efficient CRT response prediction model holds immense clinical significance in ensuring individualized medical treatment and alleviating economic burden. This study endeavors to establish a nomogram risk prediction model based on clinical features, laboratory tests, and imaging examination indicators, hoping to aid clinicians in determining optimal CRT patients and ultimately enhancing their quality of life.

## Method

### Study population

We retrospectively collected comprehensive data from 109 patients admitted to the First Affiliated Hospital with Nanjing Medical University from January 2018 to December 2022. All patients in this cohort successfully underwent CRT implantation and met the inclusion criteria: (1) preoperative New York Heart Association (NYHA) functional class II to IV; (2) completed 6-month postoperative follow-up; (3) had complete clinical data. The exclusion criteria included: (1) incomplete follow-up data or a follow-up period of fewer than 6 months; (2) upgrading of other types of pacemakers to CRT; (3) presence of other severe comorbidities such as acute coronary syndrome, malignant tumors, severe liver, and kidney failure. Patients who had improved NYHA classification by at least one level or a left ventricular ejection fraction increase of ≥ 5% compared to baseline after six months of follow-up were categorized into the CRT-responsive group [5].

### **Data source**

#### Clinical data collection

Collect clinical data of patients through outpatient and inpatient records, including the following contents: age, gender, clinical manifestations, family history, medical history, comorbidities, electrocardiogram, cardiac ultrasound, cardiac magnetic resonance, intraoperative conditions and other results.

#### Electrocardiogram examination

Using a standard 12-lead electrocardiogram (ECG) machine(FUKUDA DENSHI, FX-8322) with a paper speed of 25 mm/s and a calibration of 10 mm/mV, preoperative ECG data were collected from CRT patients, specifically focusing on the QRS duration and the QTc interval, which was calculated using the Bazett’s square root correction formula. Left ventricular high voltage was defined as Rv5 > 2.5 mV, or Rv5 + Sv1 > 4.0 mV (for males) or > 3.5 mV (for females).

#### Cardiac ultrasound examination

Using the Philips-IE33 color Doppler ultrasound diagnostic instrument with a probe frequency of 2–4 MHz, the left atrial diameter (LAD), left ventricular diastolic diameter (LVDd), left ventricular systolic diameter (LVSd), aortic root diameter (Aod), ventricular septal thickness (IVS), left ventricular posterior wall thickness (LVPW), right atrial diameter (RAD), right ventricular diastolic diameter (RVDd), shortening fraction (FS), and ejection fraction (EF) were measured before and six months post- surgery for each patient. All measurements were completed by a professional physician.

#### Cardiac magnetic resonance imaging

Cardiac magnetic resonance imaging was performed using the Siemens 3.0 T MR scanner (MAGNETOM Skyra, Siemens Healthcare, Erlangen, Germany) with multimodal imaging. All cardiac magnetic resonance images were independently evaluated by a senior cardiovascular MR diagnostic expert to obtain left ventricular functional parameters, including End-diastolic volume(EDV), End-systolic volume(ESV), Stroke volume(SV), Cardiac output(CO), Left ventricular end diastolic mass(LVMED), End-diastolic volume index(EDVI), End-systolic volume index(ESVI), Stroke volume index(SVI), cardiac index(CI), Left ventricular end diastolic mass index(LVMIED). Diffuse fibrosis was quantitatively evaluated using T1 mapping technique of cardiac magnetic resonance imaging.

### Statistical analysis

Statistical analysis was performed using SPSS 24.0 software. Quantitative data were validated for normal distribution through Kolmogorov Smirnov (K-S) test, with *P* > 0.05 indicating a normal distribution. Variables that followed a normal distribution were expressed as mean ± standard deviation (x ± s), while those did not follow normal distribution were represented by the median and interquartile range. For comparison between groups, t-tests and non-parametric rank-sum tests were employed. Categorical and ordinal data were expressed as frequencies and percentages, and χ² test was used for comparison between groups. R software version 4.2.1, along with various R packages such as “CBCgrps“ [6], “pROC“ [7], “rms”, “rmda”, and “ggplot2”, were utilized to perform nomogram, calibration plot, ROC curve, Decision Curve Analysis (DCA), and visualization analysis. The significance level was set at a two-tailed test with *P* < 0.05.

## Results

A total of 109 patients with chronic heart failure were included in this study, including 32 female patients. Among them, 69 patients had dilated cardiomyopathy, 7 had ischemic heart disease, and 33 had heart disease due to other reasons. All patients successfully received CRT implantation (Table 1). Of the 109 patients, 61 were classified as responsive, while the remaining 48 were classified as non-responsive. Baseline data before CRT implantation for both groups was presented in Table 1. There were significant differences between the two groups in terms of the proportion of LBBB, RBBB, atrioventricular block, LVDs, LVDd, IVS, EDV, ESV, EDVI, ESVI and the proportion of diffuse fibrosis (*P* < 0.05).

**Table 1:** Baseline data of patients with chronic heart failure

Variables	non-responsive group (*n* = 48)	responsive group (*n* = 61)	*P* value
Age(year)	64.1 ± 10.7	63.2 ± 11.7	0.666
Female, *n* (%)	14 (29%)	18 (30%)	0.969
Hypertension, *n* (%)	22 (46%)	26 (43%)	0.888
CHD, *n* (%)	12 (25%)	13 (21%)	0.822
Atrial fibrillation, *n* (%)	17 (35%)	17 (28%)	0.525
COPD, *n* (%)	2 (4%)	2 (3%)	0.807
Diabetes, *n* (%)	10 (21%)	9 (15%)	0.564
Chronic kidney disease, *n* (%)	5 (10%)	1 (2%)	0.085
BNP(pg/mL)	1238.5 (681, 2763.7)	1727 (912, 4847)	0.086
Electrocardiogram examination			
LBBB, *n* (%)	22 (46%)	53 (87%)	< 0.001
RBBB, *n* (%)	8 (17%)	1 (2%)	0.01
Atrioventricular Block, *n* (%)	17 (35%)	10 (16%)	0.031
Left ventricular high voltage, *n* (%)	3 (6%)	7 (11%)	0.508
QRS(ms)	169.52 ± 28.99	171.15 ± 26.17	0.762
QTc(ms)	497.25 ± 41.23	494.18 ± 35.03	0.681
Electrocardiogram examination			
Aod(mm)	29.40 ± 3.67	29.49 ± 3.22	0.885
LAD(mm)	47.06 ± 8.15	47.39 ± 6.91	0.819
LVDd(mm)	65.65 ± 8.86	72.00 ± 10.83	0.001
LVDs(mm)	55.81 ± 9.55	62.11 ± 10.63	0.002
IVS(mm)	9 (8, 11)	8 (8, 10)	0.021
LVPW(mm)	8.59 ± 1.52	8.48 ± 1.24	0.693
RAD(mm)	38.92 ± 7.07	37.84 ± 6.67	0.415
RVDd(mm)	36.96 ± 5.34	37.57 ± 7.42	0.629
FS(%)	14.9 (12.3, 17.62)	13.4 (11.5, 16.9)	0.107
EF(%)	31.15 (25.67, 36.1)	28 (24.2, 34.3)	0.086
Cardiac Magnetic Resonance Imaging			
EDV(ml)	381.5 (281.12, 497)	272 (206, 321)	< 0.001
ESV(ml)	310 (222.35, 417.08)	209 (142.8, 270)	< 0.001
SV(ml)	66.79 ± 25.00	61.09 ± 16.30	0.154
CO(l/mim)	4.37 ± 1.97	4.19 ± 1.13	0.563
LVMED(g)	206.64 ± 65.84	203.36 ± 53.11	0.78
EDVI(ml/m^2)	207.5 (162.27, 278.22)	161 (129, 190)	< 0.001
ESVI(ml/m^2)	168.55 (125.5, 249.38)	120 (88, 158)	< 0.001
SVI(ml/m^2)	38.21 ± 13.21	35.59 ± 9.00	0.223
CI(l/min/m^2)	2.50 ± 1.03	2.46 ± 0.65	0.826
LVMIED(g/m^2)	119.21 ± 31.69	118.68 ± 30.06	0.93
Diffuse fibrosis, n (%)	18 (38%)	3 (5%)	< 0.001

CHD: Coronary heart disease; COPD: Chronic obstructive pulmonary disease; LBBB: Left bundle branch block; RBBB: Right bundle branch block; Aod: inner diameter of the ascending aorta; LAD: Left atrial diameter; LVDd: left ventricular diastolic inner diameter; LVDs: left ventricular systolic inner diameter; IVS: interventricular septal thickness; LVPW: posterior wall thickness of the left ventricle; RAD: right atrial diameter; LVDd: right ventricular diastolic inner diameter; FS: fractional shortening; EF: ejection fraction; EDV: End-diastolic volume; ESV: End-systolic volume; SV: Stroke volume; CO: Cardiac output; LVMED: Left ventricular end diastolic mass; EDVI: End-diastolic volume index; ESVI: End-systolic volume index; SVI: Stroke volume index; CI: cardiac index; LVMIED: Left ventricular end diastolic mass index

All patients completed CRT implantation surgery. 43 cases in the reactive group had left ventricular electrodes placed in the left ventricular lateral vein, 18 cases in the left ventricular lateral posterior vein, 57 cases with atrial electrodes open, and 59 cases with biventricular pacing; In the non responsive group, 31 left ventricular electrodes were placed in the left ventricular lateral vein, 14 in the left ventricular lateral posterior vein, 43 with atrial electrodes open, and 45 with biventricular pacing. There was no significant difference in left ventricular electrode position, atrial electrode opening ratio, and biventricular pacing ratio between the two groups (*P* > 0.05).

**Fig. 1:**
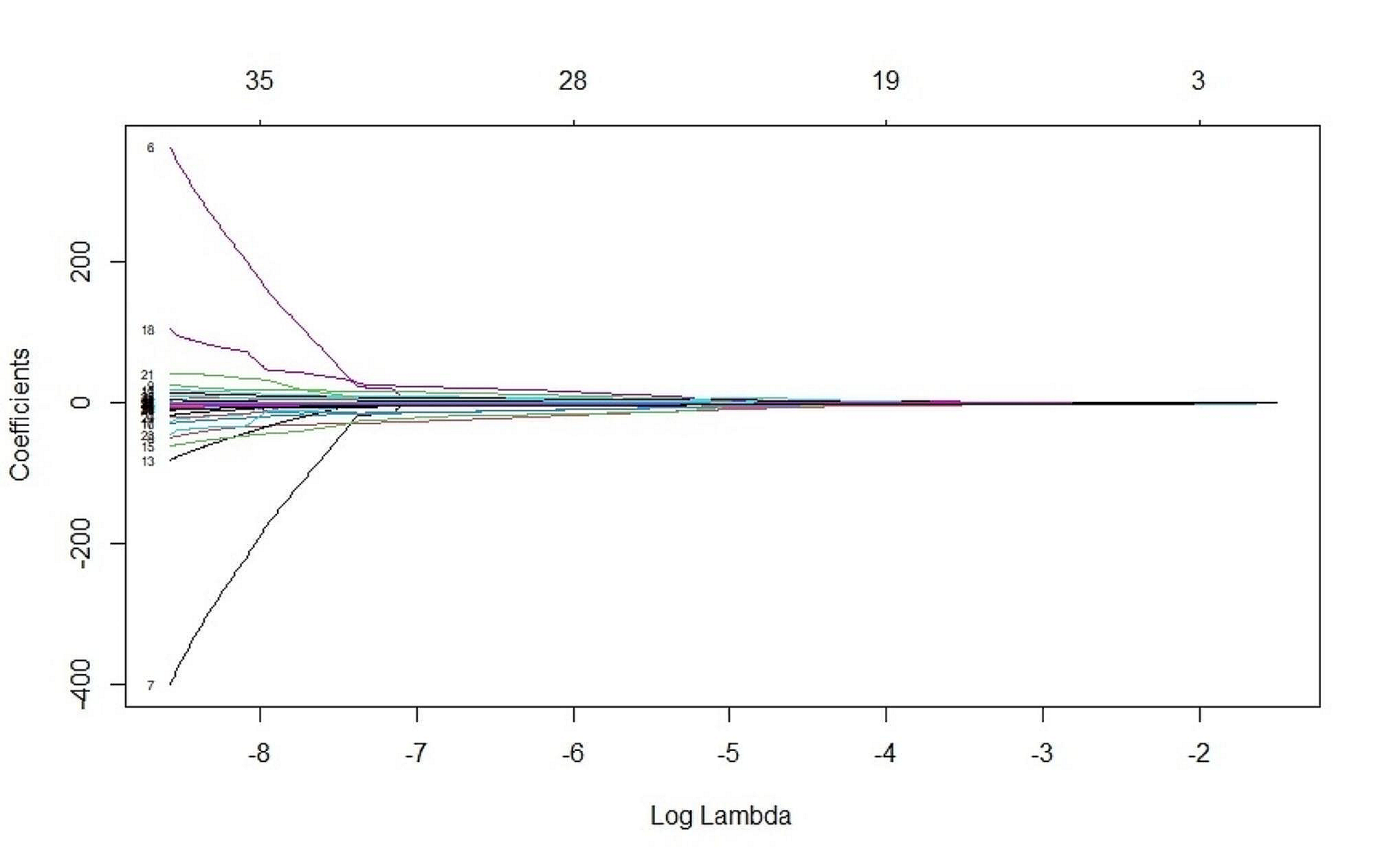
Lasso regression screening variables

**Fig. 2:**
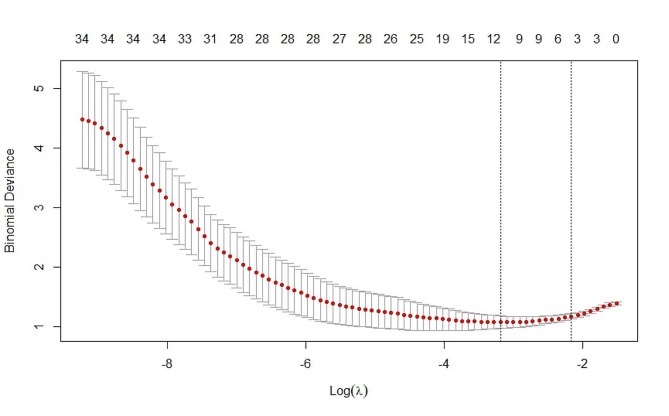
Lasso regression generates the best penalty coefficient

Using the “glmnet” package in R version 4.2.2, we perform a LASSO regression analysis to select variables. Using the CRT responsiveness of patients as the dependent variable (coded as non-responsive = 0 and responsive = 1), we considered clinical data and relevant examination indicators independent variables. Through applying the LASSO regression model, we identified three potential influencing factors that significantly affected CRT responsiveness: ESV, diffuse fibrosis, and LBBB. The results of this analysis are graphically represented in Figs. 1 and 2, which provide a clear visualization of the identified factors and their respective contributions to CRT responsiveness.

Using the CRT responsiveness of patients as the dependent variable and the influencing factors identified through LASSO regression as the independent variables, a multivariate Logistic regression analysis was conducted. As summarized in Table 2, the analysis revealed that ESV, diffuse fibrosis, and LBBB were significant independent predictors of CRT responsiveness among patients with heart failure (*P* < 0.05).

**Table 2:** Multivariate logistic regression analysis of CRT responsiveness

Variables	B	SE	Waldχ2	*P* value	OR(95%CI)
LVDd	0.0584	0.0319	1.83	0.0674	1.06(1-1.13)
ESV	-0.0097	0.0034	-2.82	0.0049	0.99(0.98-1)
Diffuse fibrosis	-2.4287	1.1417	-2.13	0.0334	0.09(0.01–0.83)
LBBB	1.8229	0.6622	2.75	0.0059	6.19(1.69–22.66)

LVDd: left ventricular diastolic inner diameter; ESV: End-systolic volume; LBBB: Left bundle branch block

**Fig. 3:**
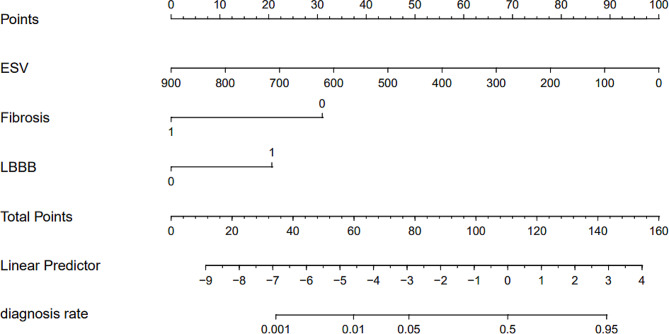
Nomogram model for predicting responsiveness to CRT therapy in patients with chronic heart failure

**Fig. 4:**
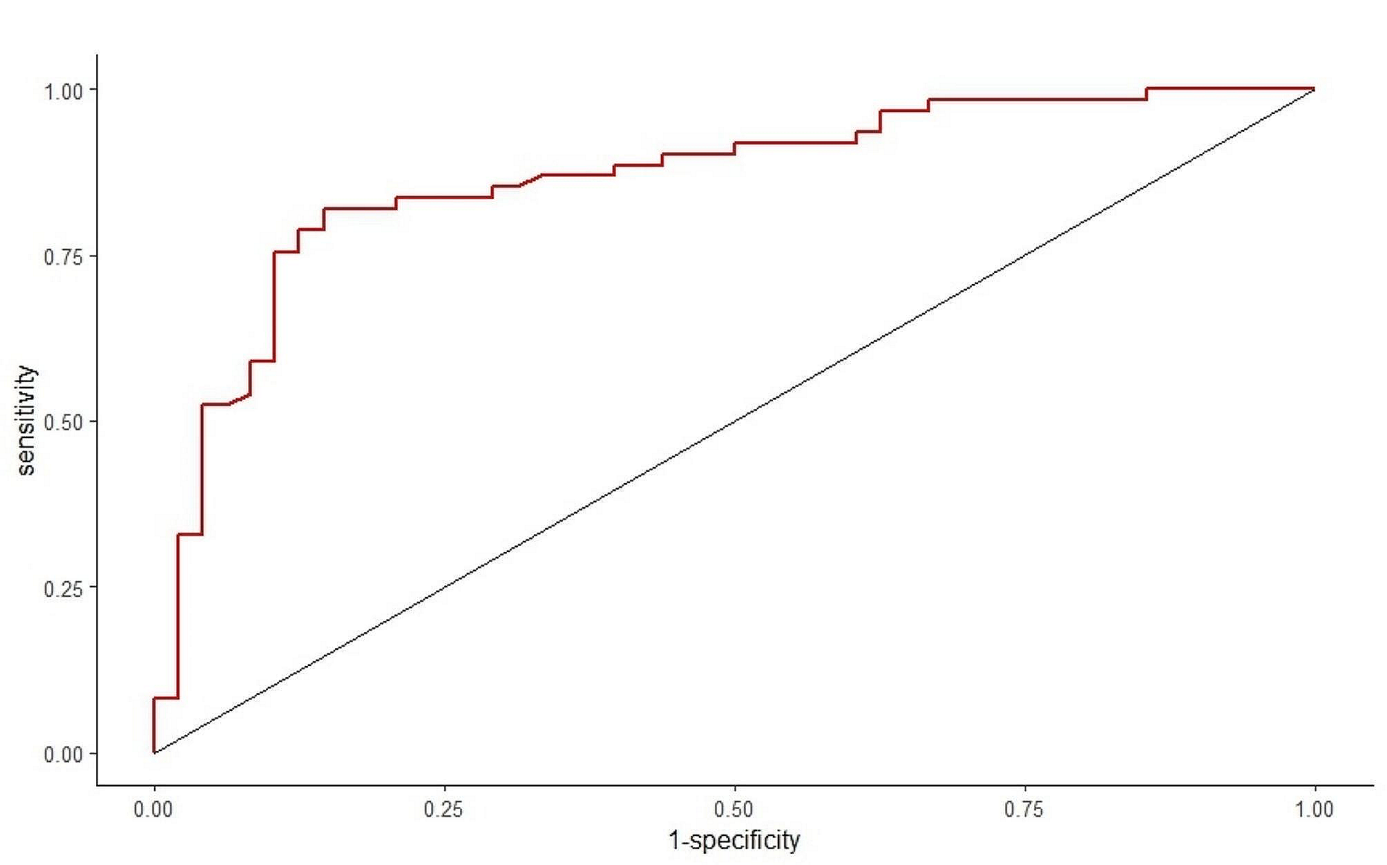
ROC curve of nomogram model predicting the occurrence of CRT response in patients with chronic heart failure

**Fig. 5:**
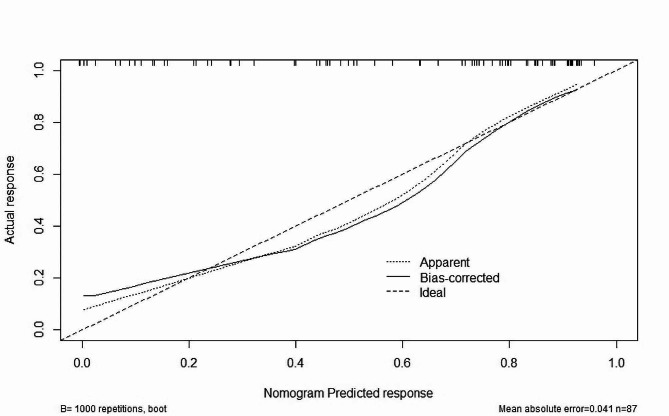
Calibration curve of nomogram model for predicting the occurrence of CRT response in patients with chronic heart failure

**Fig. 6:**
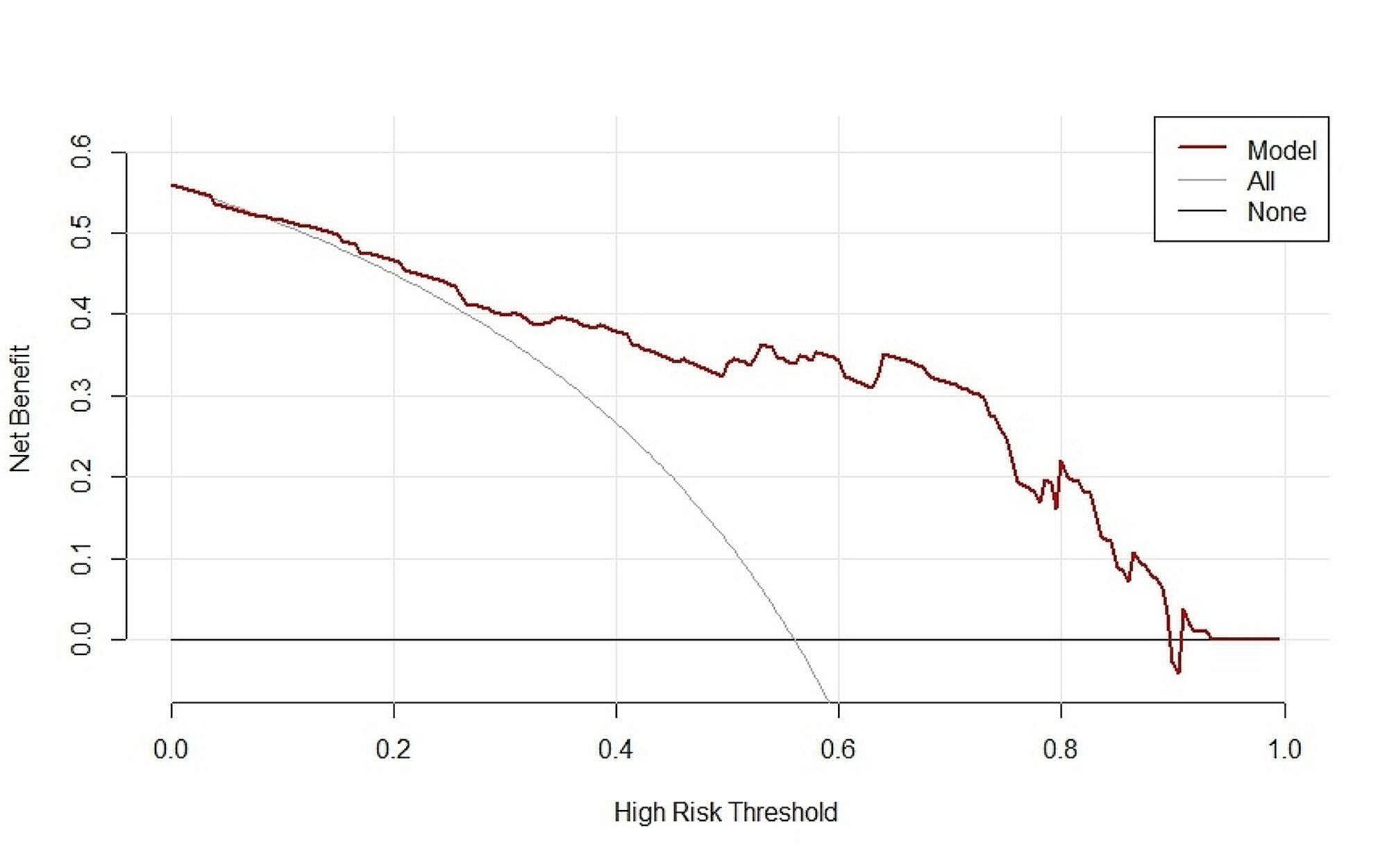
DCA decision curve of nomogram model predicting the occurrence of CRT response in patients with chronic heart failure

Based on the factors above, a nomogram model was constructed to predict the responsiveness of CRT treatment in patients with chronic heart failure (Fig. 3). ROC curve analysis revealed that the area under the curve (AUC) for the nomogram model in predicting a positive response to CRT treatment was 0.8648 (95% CI 0.7941–0.9354) (Fig. 4). Calibration curve analysis showed that the calibration curve of the nomogram model for predicting CRT responsiveness in patients with heart failure closely resembled the ideal curve (Fig. 5). DCA results suggested that the nomogram model exhibited a high clinical net benefit rate (Fig. 6).

## Discussion

In recent years, with the development of machine learning theory, machine learning algorithms have been widely used to reveal the occurrence and development of cardiovascular diseases [8, 9]. In this study, we aimed to develop and validate a nomogram model that could predict the responsiveness of CRT implantation in patients with chronic heart failure. This model was constructed based on a comprehensive analysis of clinical characteristics, laboratory tests, and imaging examination indicators. We employed the LASSO regression analysis technique to ensure our predictions’ accuracy and reliability. The method effectively compressed the patient’s multi-factorial characteristics’ regression coefficients, helping us identify the most significant predictors. This process narrowed the potential predictive variables to three key factors: ESV, diffuse fibrosis, and LBBB. This approach reduced the risk of overfitting and ensured that our logistic regression analysis was based on the most relevant variables. Subsequently, we used these three predictive variables to construct our nomogram model. The model was designed to provide a visual and quantitative tool for predicting CRT responsiveness. Through calibration curve and decision curve analysis, we found that the nomogram model exhibited excellent consistency and accuracy in predicting CRT responsiveness. Importantly, all the indicators used to construct this model are easily obtainable in clinical practice. This makes our nomogram a convenient and practical tool for clinicians to use in assessing the potential benefits of CRT in their patients with chronic heart failure.

ESV refers to the minimum volume of the ventricle after contraction. In clinical practice, the stroke volume is usually calculated by calculating the difference between EDV and ESV. This value is of great significance for evaluating left ventricular function, and a decrease in ESV is also an important indicator for evaluating CRT responsiveness. Chronic heart failure is a progressive disease. CRT can alleviate or reverse ventricular remodeling when the ventricular function is impaired within a specific range. At this time, the ESV is still within normal range, but when it reaches an irreversible stage, the heart has been severely damaged, and even through CRT means, there will be no significant effect. At this time, the ESV increases significantly and cannot compensate. The clinical course of heart failure is determined by cardiac remodeling [10]. A decrease in left ventricular ESV means reverse remodeling. EF, as an indicator of left ventricular systolic function, is less suitable as a surrogate indicator, possibly because EF depends more on left ventricular end-diastolic volume and heart rate [11]. Liu Liwen et al. proposed that ESV is an independent risk factor for CRT non-responsiveness [12], and Uhm et al. also demonstrated that ESV can predict the 1-year stratified clinical composite endpoint in patients undergoing cardiac resynchronization therapy [13].

Myocardial fibrosis serves as a crucial indicator of myocardial injury and dysfunction, characterized primarily by the proliferation of fibroblasts and excessive deposition of collagen in the extracellular matrix of normal myocardial tissue. This pathological condition can alter the mechanical and electrophysiological functions of the heart, potentially leading to heart failure and even sudden cardiac death. Myocardial fibrosis plays a pivotal role in the progression of heart failure, as it not only accelerates deterioration but also serves as a crucial marker for assessing myocardial remodeling. Cardiac magnetic resonance (CMR) imaging [14] has emerged as a powerful tool for detecting myocardial fibrosis’s presence, location, and extent within the ventricle. This non-invasive technique offers insights into the microstructure of the heart, providing clinicians with valuable information for diagnosis and treatment planning. Extensive research has demonstrated a close association between the morphology and phenotype of myocardial delayed enhancement observed on CMR and patient prognosis. Specifically, patients exhibiting myocardial delayed enhancement tend to have a poorer clinical outcome. Furthermore, the extent of delayed enhancement appears to correlate with the severity of the prognosis, with more diffuse enhancement indicating a worse prognosis. The degree of diffuse myocardial fibrosis assessed by CMR provides insights into the extent of myocardial remodeling. A lower degree of fibrosis suggests less severe remodeling, which may indicate a more favorable response to therapeutic interventions. In the context of CRT, the assessment of myocardial fibrosis becomes particularly relevant. CRT involves the placement of electrodes within the left ventricle to restore coordinated contraction. The ability of these pulses to effectively stimulate viable myocardial tissue and restore contractile function in previously dysfunctional myocardium is influenced by the degree of fibrosis [15]. Therefore, assessing myocardial fibrosis and its extent using CMR holds significant prognostic value for patients undergoing CRT. By understanding the extent and distribution of fibrosis, clinicians can gain insights into the potential benefits of CRT and tailor treatment strategies accordingly. This personalized approach to therapy optimization can lead to improved clinical outcomes and a better quality of life for patients with heart failure.

LBBB serves as a pivotal predictor of mortality among heart failure patients who have not undergone resynchronization therapy and exhibit EF of 39% or below [16]. However, the situation reverses in patients selected to receive Cardiac Resynchronization Therapy (CRT), with LBBB often associated with more favorable clinical outcomes [17]. Research conducted by Ginks et al. [18]. has identified two distinct electrical activity patterns within LBBB. Type I is characterized by a uniform spread of electrical excitation from the interventricular septum to the lateral wall, indicating a more coordinated electrical activity. Conversely, Type II exhibits a “U-shaped” spread due to a linear functional block between the septum and lateral wall, leading to delayed trans-septal conduction time. Patients with Type I electrical activity patterns tend to respond better to CRT. Multiple studies have also confirmed that LBBB is an independent predictor of response to CRT. Killu et al. found that LBBB can increase the likelihood of an super-response(SR) to CRT [19]. Rickard J et al. systematically analyzed multiple studies from 1995 to 2014 to identify variables associated with “response” to CRT, and the results suggested that LBBB is significantly associated with improved prognosis after CRT implantation [20]. Similarly, Xiao Pei-Lin et al. developed a nomogram model based on clinical variables, which indicated that patients without intrinsic LBBB are less likely to benefit from CRT [21]. Current research has introduced an alternative pacing strategy for the conduction system, known as left bundle branch pacing (LBBP), which aims to improve LVEF by correcting LBBB with a low and stable threshold. A novel prospective, multicenter, case-control study suggests that upgrading to LBBP is feasible and effective for patients who are unresponsive to cardiac resynchronization therapy (CRT), indicating the potential of LBBP as an alternative treatment to CRT [22]. A separate prospective, multicenter observational study comparing LBBP, left ventricular septal pacing, and biventricular pacing revealed that LBBP outperforms both left ventricular septal pacing and biventricular pacing in improving heart failure (HF)-related hospitalizations and all-cause mortality [23]. Consequently, we can anticipate that LBBP has the potential to become a reasonable and promising pacing strategy.

Our study, while offering valuable insights, has its limitations. Firstly, the study subjects’ relatively small sample size may affect the research findings’ generalizability. A larger sample size likely enhanced the representativeness of the results. Secondly, the retrospective nature of the study introduces potential biases. The selection of indicators was constrained by the available data, which may cause selection bias. To mitigate this, future prospective studies are warranted to validate the predictive model obtained in this study. Furthermore, the follow-up period for the patients was relatively short. Long-term follow-up data would provide a more comprehensive assessment of CRT effectiveness. This is particularly important given the chronic nature of heart failure and the potential for long-term changes in patient outcomes.

In conclusion, our study has successfully constructed a nomogram prediction model for CRT treatment in chronic heart failure patients. The model, which incorporates left ventricular end-systolic volume, diffuse myocardial fibrosis, and left bundle branch block, exhibits strong consistency, accuracy, and clinical utility. It effectively predicts patient response to CRT treatment, aiding clinicians in predicting response rates and ultimately reducing the burden on the healthcare system.

## Data Availability

The datasets used and/or analysed during the current study available from the corresponding author on reasonable request.
